# Does the Short-Term Effect of Air Pollution Influence the Incidence of Spontaneous Intracerebral Hemorrhage in Different Patient Groups? Big Data Analysis in Taiwan

**DOI:** 10.3390/ijerph14121547

**Published:** 2017-12-10

**Authors:** Ting-Ying Chien, Hsien-Wei Ting, Chien-Lung Chan, Nan-Ping Yang, Ren-Hao Pan, K. Robert Lai, Su-In Hung

**Affiliations:** 1 Innovation Center for Big Data and Digital Convergence, Yuan Ze University, Taoyuan 320, Taiwan; tinin@saturn.yzu.edu.tw (T.-Y.C.); clchan@saturn.yzu.edu.tw (C.-L.C.); 2 Department of Computer Science and Engineering, Yuan Ze University, Taoyuan 320, Taiwan; ting.ns@gmail.com (H.-W.T.); pan@51donate.com (R.-H.P.); 3 Department of Neurosurgery, Taipei Hospital, Ministry of Health and Welfare, New Taipei City 242, Taiwan; 4 Department of Information Management, Yuan Ze University, Taoyuan 320, Taiwan; 5 Community Medicine Research Center, National Yang-Ming University, Taipei 112, Taiwan; yang.nanping@gmail.com; 6 Department of Surgery, Keelung Hospital, Ministry of Health and Welfare, Keelung City 201, Taiwan; 7 Department of Information Management, Tunghai University, Taichung 407, Taiwan; 8 Center of Quality and Patient Safety Management, Taipei Hospital, Ministry of Health and Welfare, New Taipei City 242, Taiwan; dear.selena@gmail.com; 9 Department of Business Administration, National Taipei University, Taipei 237, Taiwan

**Keywords:** air pollution, big data, meteorological factors, spontaneous intracerebral hemorrhage

## Abstract

Spontaneous intracerebral hemorrhage (sICH) has a high mortality rate. Research has demonstrated that the occurrence of sICH is related to air pollution. This study used big data analysis to explore the impact of air pollution on the risk of sICH in patients of differing age and geographic location. 39,053 cases were included in this study; 14,041 in the Taipei region (Taipei City and New Taipei City), 5537 in Taoyuan City, 7654 in Taichung City, 4739 in Tainan City, and 7082 in Kaohsiung City. The results of correlation analysis indicated that there were two pollutants groups, the CO and NO_2_ group and the PM_2.5_ and PM_10_ group. Furthermore, variations in the correlations of sICH with air pollutants were identified in different age groups. The co-factors of the influence of air pollutants in the different age groups were explored using regression analysis. This study integrated Taiwan National Health Insurance data and air pollution data to explore the risk factors of sICH using big data analytics. We found that PM_2.5_ and PM_10_ are very important risk factors for sICH, and age is an important modulating factor that allows air pollutants to influence the incidence of sICH.

## 1. Introduction

It is well-known that pollution influences health, including cardiovascular diseases, cerebrovascular diseases, pulmonary diseases, and some other diseases [1,2,3]. Evidence has indicated that air pollutants including particulate matter (PM), ozone (O_3_), nitrogen dioxide (NO_2_), sulfur dioxide (SO_2_) and carbon monoxide (CO) influence health [4,5]. Spontaneous intracerebral hemorrhage (sICH) accounts for 10–35% of stroke patients; the incidence ranges from 10 to 60 cases per 100,000 populations per year, with a high mortality rate [6,7,8]. Research has indicated that air pollution is correlated with the incidence of and hospital admissions due to stroke, but different conclusions have been reached by different research groups with regards to the correlations between sICH and air pollutants [4,9,10,11,12]. The Taiwan National Health Insurance Research Database (NHIRD) is a useful longitudinal dataset for financial and epidemiological research. Many studies have used the NHIRD for exploration of medical issues [8,13,14,15]; however, few studies have integrated the NHIRD with other datasets. The dataset used in this study included outpatient and inpatient claims data, with detailed longitudinal information for each visit/stay [16]. This study integrated the NHIRD and governmental open data using big data analysis methods and evaluated the correlations between air pollutants and sICH in Taiwanese patients in short-term effect (exposure to air pollution over one to 24 h). 

## 2. Materials and Methods

### 2.1. Data Sources

This study integrated the National Health Insurance Research Database (NHIRD), the household registration database of the Department of Household Registration, the 2010 population and housing census, and the air pollutants data derived from the government open data platform in Taiwan with big data analytics systems using the platform of the Innovation Center for Big Data and Digital Convergence, Yuan Ze University [16,17]. 

### 2.2. Data Protection and Permission

The personal information of all subjects was encrypted using a double scrambling protocol for research purposes to protect patient privacy. All researchers who wish to use the NHIRD and its data subsets are required to sign a written agreement declaring that they have no intention of obtaining information that could potentially violate the privacy of patients or care providers. This study was approved by the Institutional Review Board (IRB) of Taipei Hospital (IRB Approval Number: TH-IRB-0015-0003), and the protocol was evaluated by the National Health Research Institutes (NHRI), which consented to this planned analysis of the NHIRD (Agreement Number: NHIRD-104-183).

### 2.3. Data Management

**Figure 1:**
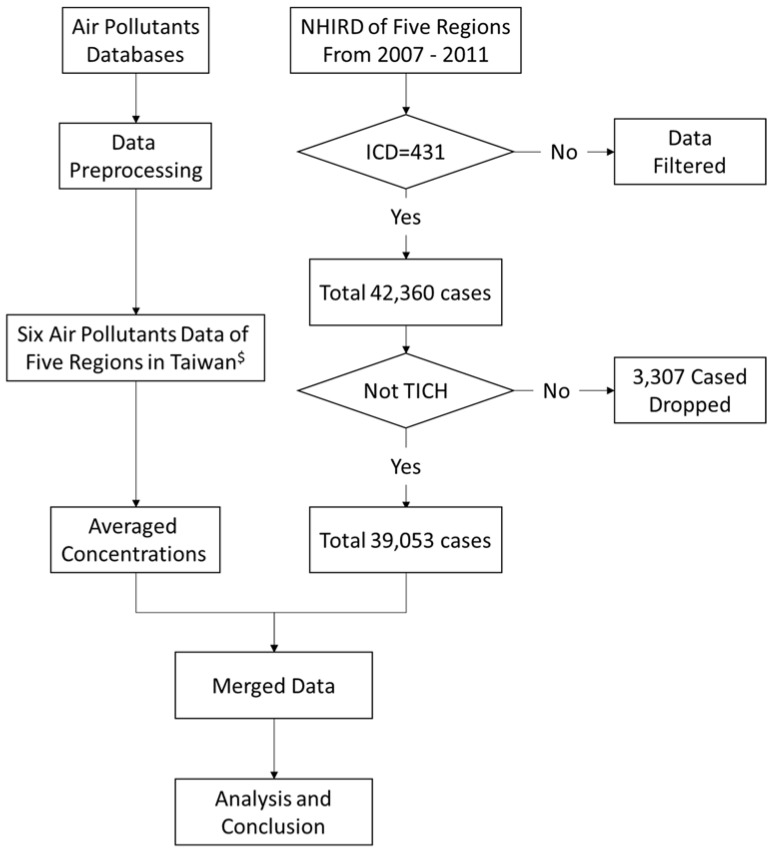
Flow chart of data management for study of sICH patients in five regions of Taiwan. ^$^ The five regions were the Taipei region (Taipei City and New Taipei City), Taoyuan City, Taichung City, Tainan City and Kaohsiung City. NHIRD: National Health Insurance Research Database; ICD: International Classification of Diseases; TICH: traumatic intracranial hemorrhage; sICH: Spontaneous intracerebral hemorrhage.

The inclusion criterion for patients in this research was first-attack sICH, identified by a principal diagnosis code of the International Classification of Diseases 9th version (ICD-9) of 431. The five regions analyzed were the Taipei region (Taipei City and New Taipei City), Taoyuan City, Taichung City, Tainan City and Kaohsiung City. In total, 42,360 sICH cases were registered from 2007 to 2011 in the five regions. Patients who were admitted due to traumatic intracranial hemorrhage (TICH) (ICD-9 codes 800.00 to 804.99, 850.00 to 854.19, 959.01, and 959.09) were excluded (3307 cases) [8], and the remaining 39,053 cases were then analyzed in this study. Data of the six air pollutants extracted from the Taiwan government open data platform, were also cleaned and merged, including data for carbon monoxide (CO), nitrogen dioxide (NO_2_), ozone (O_3_); particulate matter (PM) 10 μm and 2.5 μm, and sulfur dioxide (SO_2_). Because of certain sensors were broken, there were some missing data. To manage the missing data, we used linear interpolation method, and then the average concentrations of each air pollutant in the 5 regions were calculated as the daily concentrations. These data were merged with observed date of each air pollutant and patients’ admission date. Finally, we analyzed those dataset (Section 2.4). A flow chart of data management in this study is as shown in Figure 1. The cut-off values indicating abnormal pollution levels, mean concentrations, and number of days on which the levels of pollutants exceeded normal levels in the five regions were also collected. 

### 2.4. Statistics

Patient characteristics including gender, age, incidence of sICH, % of patients on a low income, Charlson Comorbidity Index (CCI) [18,19] and total hospital length of stay (LOS) were collected by region. The Pearson correlation coefficient and stepwise regression statistics were used to examine the relationships between data. In this study, the degree of correlation was defined as follows: high correlation was defined as a correlation coefficient larger than 0.6; moderate correlation was defined as a correlation coefficient between 0.3 and 0.6; and low correlation was defined as a correlation coefficient lower than 0.3. Statistical analysis was conducted using SPSS version 19.0 (SPSS Inc., Chicago, IL, USA). The big data analysis and visualization tools were constructed by the Innovation Center for Big Data and Digital Convergence, Yuan Ze University [20]. Statistical significance was defined as *p* < 0.05. 

## 3. Results

The correlations between the incidence of sICH and air pollutant levels in five regions of Taiwan were examined. 39,053 cases were included in this study, 14,041 in the Taipei region (Taipei City and New Taipei City), 5537 in Taoyuan City, 7654 in Taichung City, 4739 in Tainan City and 7082 in Kaohsiung City. The lowest incidence of sICH was found in the Taipei region (216.4 cases per 100,000 populations per year). The incidences of sICH in both Taoyuan City (280.0 cases per 100,000 populations per year) and Taichung City (290.4 cases per 100,000 populations per year) were higher than those of the other cities for both male and female patients. There were no significant differences in terms of mean age or Charlson Comorbidity Index (CCI) between patients from the different regions (Table 1). 

The cut-off points indicating abnormal levels of carbon monoxide (CO) and nitrogen dioxide (NO_2_) were 250 ppb and 35 ppm, respectively. Neither the mean concentrations nor the number of days on which the criteria for normality were exceeded for CO and NO_2_ were observed to be abnormal in any of the 5 regions/cities investigated in this study. The abnormal level cut-off point for ozone (O_3_) was 120 ppb. Although the mean concentration of O_3_ was within the normal limit, there were still more than 300 days within the 5-year study period on which the cut-off level was exceeded in all regions assessed in this study. The city with the lowest level of O_3_ was Taoyuan City, the cut-off level being exceeded on only 356 days during the 5-year study period. The city with the highest level of O_3_ was Kaohsiung City, for which 1035 days in the 5-year study period exceeded the level indicating abnormal O_3_ pollution. The cut-off level indicating an abnormal particulate matter (PM) 10 μm (PM_10_) pollution level was 125 μg/m^3^. It was found that the mean concentration of PM_10_ was within the normal limit in all 5 regions/cities; however, in Tainan City (127 days in five years) and Kaohsiung City (183 days in five years), the cut-off limit for the PM_10_ concentration was exceeded on more than 100 days within the 5-year study period. The cut-off level indicating an abnormal PM _2.5_ μm pollution level was 35 μg/m^3^. The records for the Taipei region (26.8 μg/m^3^) and Taoyuan City (28.0 μg/m^3^) showed that these regions were within the normal limits in terms of the mean concentration of PM_2.5_. However, in 3 regions/cities, the cut-off level indicating abnormal pollution was exceeded on more than 730 days (2 years) within the 5-year study period. The levels in Taipei and Taoyuan City exceeded the cut-off point on fewer days (i.e., less than 2 years), but the normal range of PM_2.5_ was still exceeded on more than 400 days. The cut-off level indicating an abnormal amount of sulfur dioxide (SO_2_) was 100 ppb. The mean concentrations of SO_2_ were within the normal limit in all 5 regions/cities (Table 2).

Regarding correlations between air pollutants, two groups were found to have extremely high correlations: the CO and NO_2_ group (correlation coefficient = 0.939, *p* < 0.01) and the PM_10_ and PM_2.5_ group (correlation coefficient = 0.969, *p* < 0.001). All other pollutants had high/moderate correlations with PM_10_ and PM_2.5_. CO had moderate correlations with PM_10_ (correlation coefficient = 0.399, *p* < 0.01) and PM_2.5_ (correlation coefficient = 0.463, *p* < 0.001). NO_2_ had high correlations with PM_10_ (correlation coefficient = 0.610, *p* < 0.001) and PM_2.5_ (correlation coefficient = 0.637, *p* < 0.001) and a moderate correlation with SO_2_ (correlation coefficient = 0.422, *p* < 0.001). O_3_ had moderate correlations with PM_10_ (correlation coefficient = 0.372, *p* < 0.01) and PM_2.5_ (correlation coefficient = 0.343, *p* < 0.01). SO_2_ had moderate correlations with PM_10_ (correlation coefficient = 0.550, *p* < 0.001) and PM_2.5_ (correlation coefficient = 0.521, *p* < 0.001) (Table 3).

Variations in the correlations of air pollutants with sICH were observed between different age groups. No correlations between air pollutants and the incidence of sICH were observed in patients under 25 years of age. NO_2_ (correlation coefficient = 0.341, *p* < 0.01) and SO_2_ (correlation coefficient = 0.296, *p* < 0.05) were correlated with the incidence of sICH in patients aged between 25 and 44 years. NO_2_ (correlation coefficient = 0.333, *p* < 0.01), PM_10_ (correlation coefficient = 0.629, *p* < 0.001) and PM_2.5_ (correlation coefficient = 0.625, *p* < 0.001) were correlated with the incidence of sICH in patients aged between 45 and 64 years. NO_2_ (correlation coefficient = 0.311, *p* < 0.05), PM_10_ (correlation coefficient = 0.689, *p* < 0.001) and PM_2.5_ (correlation coefficient = 0.695, *p* < 0.001) were correlated with the incidence of sICH in patients aged between 65 and 79 years. CO (correlation coefficient = 0.383, *p* < 0.01), NO_2_ (correlation coefficient = 0.473, *p* < 0.001), PM_10_ (correlation coefficient = 0.456, *p* < 0.001) and PM_2.5_ (correlation coefficient = 0.445, *p* < 0.001) were correlated with the incidence of sICH in patients over 80 years of age (Table 3).

In this study, the co-factors related to the influences of air pollutants on the incidence of sICH in different age groups were evaluated using regression analysis. For the extremely high correlations of two groups described above. We erased the PM_10_ and CO. Three models were used to examine the correlations of the monthly incidence of sICH with air pollutants. Model 1 included only PM_2.5_; the adjusted R^2^ of Model 1 was 0.417. Although the two other models also had satisfactory adjusted R^2^ values (0.459 and 0.490), it was not logical that the two other factors (O_3_ and SO_2_) had a negative influence, as this would mean that the higher the concentration of these air pollutants, the lower the monthly incidence of sICH. In addition, the F values of the change in R^2^ were lower than that of Model 1. Therefore, Models 2 and 3 were suspended (Table 4).

In terms of age, there were no significant correlations between the air pollutants and the monthly incidence of sICH in patients under 25 years of age, and only one factor (NO_2_) that was included in the regression model was significantly correlated with the monthly incidence of sICH in patients aged between 25 and 44 years; however, the adjusted R^2^ (0.101) was too low to be accepted. Regarding the regression models that included patients aged between 45 and 64 years, the same problems were experienced for Models 2, 3 and 4, in that O_3_, SO_2_ and NO_2_ were shown to have negative influences. Only Model 1, which contained only PM_2.5_, was acceptable, with a satisfactory adjusted R^2^ (0.474). For analysis of patients aged over 80 years, only NO_2_ was included in the regression model, the adjusted R^2^ of which was 0.211 (Table 5).

## 4. Discussion

In recent studies, it has been found that PM_2.5_ and PM_10_ have great impacts on human health, especially PM_2.5_ [21]. However, the conclusions reached with regards to the correlations of ambient PM_2.5_ and PM_10_ concentrations with the risk of sICH were inconsistent [4,9,22,23,24]. Tsai et al. [4] concluded that air pollutants (PM_10_, O_3_, NO_2_, SO_2_ and CO) are highly correlated with sICH admissions in warm weather (environment temperature > 20 °C). Xiang et al. [9] evaluated air pollutants NO_2_, SO_2_ and PM_10_, and concluded that no significant correlations existed between the air pollutants and sICH admissions in warm weather, but reported that NO_2_ is significantly associated with stroke during cold weather. A meta-analysis study of ambient particulate matter levels concluded that PM_2.5_ and PM_10_ have no influence on the risk of sICH in patients of any age [22]. As the concentrations of PM_2.5_ and PM_10_ were found to be very highly correlated in the present study, it was concluded that PM_2.5_ and PM_10_ are important risk factors for sICH. In addition, again owing to there being significant correlations between PM_10_ and PM_2.5_, a regression model was conducted in this study to predict the correlation between the number of cases of sICH per month and PM_2.5_ (R^2^ = 0.417). 

Age was found to be an important modulating factor of the effect of air pollutants on the incidence of sICH. Different levels of influence of air pollution on the risk of sICH were observed in different age groups. We found high correlations between sICH and PM_10_, PM_2.5_ and NO_2_ in the middle-aged and elderly patient groups. The concentration of NO_2_ was also correlated with the incidence of sICH in patients aged between 25 and 44 years in this study. We adjusted the factors using regression analysis in order to prevent internal correlations between factors. Previous research has resulted in inconsistent conclusions regarding the influence of NO_2_ on the risk of sICH [4,10,11,12,23,24]. In this study, when other air pollutant factors were controlled, NO_2_ was found to influence the risk of sICH in patients aged over 80 years (R^2^ = 0.211). We also found that the ambient levels of air pollutants did not influence the risk of sICH in younger patients (<44 years of age). PM_2.5_ and PM_10_ were found to be very important risk factors for sICH in middle-aged and elderly sICH patients (45–79 years of age). 

Previous study has shown that air pollutants are highly correlated with the incidence of stroke in Taiwan [4,23,24]. In this study, it was found that on no days did the levels of CO, NO_2_ and SO_2_ exceed normal levels in any of the regions examined (Table 2). We believe that this is good evidence of pollution control by the Taiwan government in the modern era. However, we found that NO_2_ pollution was still highly correlated with the incidence of sICH, and the number of days on which the levels of PM_10_, PM_2.5_ and O_3_ exceeded normal levels remained high. Moreover, the mean concentration of PM_2.5_ in some cities was higher than the cut-off level, indicating abnormally high levels. This raises the question as to whether the cut-off levels for air pollutants at which the level is considered abnormal are too high to prevent influences of the pollutants on the incidences of diseases. The air pollution control policies and cut-off levels may therefore need to be readjusted following further in-depth research.

We found most of the air pollutants had middle to high correlation, except O_3_. The possible reason was that O_3_ is formed from hydrocarbons and nitrogen oxides reactive with sunlight and it may spread to many kilometers by wind, but the others pollutants are produced by car engines or by industrial operations. Most of the air pollutants in this study were correlated with PM_10_ and PM_2.5_. Owing to most ambient particulate matter being a heterogeneous mixture of various compounds, such as organic and elemental carbon, metals, sulfates, nitrates, and some microorganisms [22], PM_2.5_ and PM_10_ are expected to be highly correlated with the levels of other pollutants. We therefore adjusted these correlations using regression analysis. 

The limitations of this study included that there were differences in the geographic positioning of air pollutant detection stations and the locations of sICH patients when they suffered an attack, and the time frames of pollutant measurement and the occurrence of sICH also differed. Other researchers also identified a time lag [23], in that patients were perhaps not sent to hospital immediately after suffering sICH.

## 5. Conclusions

As sICH is an emergency condition for which most patients are sent to the ER at once, the results of this study can be considered reliable. This is a pilot study for the fast reaction effect of these pollution factors on sICH. We found of the air pollutions still influence the incidence of sICH. This study found that NO_2_ pollution was still highly correlated with the incidence of sICH, and the number of days on which the levels of PM_10_, PM_2.5_ and O_3_ exceeded normal levels remained high. In addition, age was found to be an important modulating factor of the effect of air pollutants on the incidence of sICH. There are high correlations between sICH and PM_10_, PM_2.5_ and NO_2_ in the middle-aged and elderly patient groups. Furthermore, PM_2.5_ and PM_10_ were found to be very important risk factors for sICH in middle-aged and elderly sICH patients. The reason is still unclear and will need to be further investigated. In the future, laboratory data and temperature may be included, and multivariate analysis can be used in order to detect the influences of air pollutants more precisely. Air pollution control policies and cut-off levels may therefore need to be readjusted following further in-depth research. 

## Figures and Tables

**Table 1:** Data of sICH patients in five regions of Taiwan (2007–2011).

Region	Taipei	Taoyuan	Taichung	Tainan	Kaohsiung
^#^ Resident population	6,709,982	2,190,342	2,731,056	1,840,257	2,777,384
Male	3,252,868	1,107,819	1,353,789	915,361	1,381,183
Female	3,457,114	1,052,523	1,377,267	924,896	1,396,201
Total case number	14,041	5537	7654	4739	7082
Female ratio (%)	38.0%	36.4%	36.9%	37.3%	37.4%
^¶^ Incidence of sICH	216.4	280.0	290.4	252.9	255.6
Male	272.6	352.5	367.4	314.0	318.2
Female	161.9	206.0	213.6	190.6	192.3
Mean age	61.9 (16.6)	60.4 (16.7)	61.6 (15.9)	62.6 (15.4)	61.5 (15.6)
Male	60.1 (16.1)	59.0 (16.4)	59.7 (15.5)	60.7 (14.8)	59.6 (15.0)
Female	64.8 (17.1)	62.8 (17.0)	64.7 (16.2)	65.8 (15.8)	64.6 (16.0)
Low-income cases (%)	250 (1.8%)	122 (2.2%)	65 (0.8%)	60 (1.3%)	83 (1.2%)
^&^ CCI					
0 (%)	6660 (47.4%)	2690 (48.6%)	4015 (52.5%)	2329 (49.1)	3476 (49.1%)
1 (%)	3834 (27.3%)	1492 (26.9%)	2007 (26.2%)	1361 (28.7%)	1829 (25.8%)
2 (%)	2356 (16.8%)	945 (17.1%)	1118 (14.6%)	723 (15.3%)	1175 (16.6%)
≥3 (%)	1191 (8.5%)	410 (7.4%)	514 (6.7%)	326 (6.9%)	602 (8.5%)
Total LOS	17.3 (14.8)	14.8 (13.4)	15.8 (13.7)	13.9 (11.9)	14.0 (11.5)

^#^ The resident population in this study was calculated as the permanent resident population using data from 2010. LOS: length of stay. ^¶^ Incidence was defined as cases per 100,000 populations per year. ^&^ CCI: Charlson Comorbidity Index.

**Table 2:** Mean concentrations of pollutants in different areas and number of days on which the normal levels were exceeded.

Air Pollutant	CO	NO_2_	O_3_	PM_10_	PM_2.5_	SO_2_
Abnormal criteria	35 ppm	250 ppb	120 ppb	125 μg/m^3^	35 μg/m^3^	100 ppb
	(30.5 ppm) ^ψ^	(1250 ppb)	(405 ppb)	(425 μg/m^3^)	(250.5 μg/m^3^)	(650 ppb)
	8-h	1-h	1-h	24-h	24-h	24-h
Mean concentration						
Taipei	0.6 (0.1)	21.1 (3.2)	28 (5.2)	46.8 (9.6)	26.8 (5.3)	3.8 (0.5)
Taoyuan	0.5 (0.1)	19.4 (2.9)	28.5 (5.3)	56.5 (11.1)	28.0 (6.1)	5.6 (0.4)
Taichung	0.5 (0.1)	18.8 (4)	28.1 (5.4)	58.5 (13.5)	35.6 (8.4)	3.5 (0.3)
Tainan	0.4 (0.1)	15.3 (4.7)	30.9 (6.5)	73.6 (23.7)	39.2 (12.5)	4.2 (0.4)
Kaohsiung	0.5 (0.2)	20.3 (6.8)	29.9 (6.6)	77.3 (27.5)	44.9 (16.4)	7.2 (1.2)
^#^ Days on which normal level was exceeded						
Taipei	0 (0) ^ξ^	0 (0)	153 (0)	13 (1)	428 (0)	0 (0)
Taoyuan	0 (0)	0 (0)	135 (0)	31 (1)	465 (0)	0 (0)
Taichung	0 (0)	0 (0)	393 (0)	32 (1)	818 (0)	0 (0)
Tainan	0 (0)	0 (0)	566 (0)	127 (1)	988 (0)	0 (0)
Kaohsiung	0 (0)	0 (0)	647 (0)	183 (1)	1159 (0)	0 (0)

^ψ^ Abnormal criteria of United States. ^#^ Number of days on which the standard cut-off value was exceeded from 2007 to 2011 (1827 days in total). ^ξ^ Number of days on which the United State standard cut-off value was exceeded from 2007 to 2011. Carbon monoxide (CO); nitrogen dioxide (NO_2_); ozone (O_3_); particulate matter (PM); sulfur dioxide (SO_2_).

**Table 3:** Correlations between air pollutant levels and incidence of sICH in different age groups.

Air Pollutant	CO	NO_2_	O_3_	PM_10_	PM_2.5_	SO_2_
Standard values	35 ppm	250 ppb	120/h	125 μg/m^3^	35 μg/m^3^	100 ppb
Correlation between air pollutants						
CO	1					
NO_2_	0.939 **	1				
O_3_	0.034	0.071	1			
PM_10_	0.399 **	0.610 ***	0.372 **	1		
PM_2.5_	0.463 ***	0.637 ***	0.343 **	0.969 ***	1	
SO_2_	0.239	0.422 ***	0.085	0.550 ***	0.521 ***	1
All patients	0.205	0.415 ***	0.013	0.658 ***	0.654 ***	0.196
<24 y/o ^ψ^	−0.015	−0.010	−0.056	−0.212	−0.226	−0.035
25–44 y/o	0.197	0.341 **	−0.163	0.197	0.180	0.296 *
45–64 y/o	0.124	0.333 **	0.033	0.629 ***	0.625 ***	0.245
65–79 y/o	0.114	0.311 *	0.096	0.689 ***	0.695 ***	0.110
≥80 y/o	0.383 **	0.473 ***	−0.053	0.456 ***	0.445 ***	−0.004

* *p* < 0.05, ** *p* < 0.01, *** *p* < 0.001. Carbon monoxide (CO); nitrogen dioxide (NO_2_); ozone (O_3_); particulate matter (PM); sulfur dioxide (SO_2_). ^ψ^ y/o: Abbreviation of years old.

**Table 4:** Regression models used to examine the correlations of the monthly incidence of sICH with air pollutants.

Models	Constant	PM_2.5_	O_3_	SO_2_	Adj. R^2^	F for Change in R^2^
Model 1			X	X	0.417	43.263 **
B	15.127 ***	0.166 ***				
SE B		0.654				
Model 2				X	0.459	5.488 *
B	18,171 ***	0.187 ***	−0.130 *			
SE B		0.735	−0.239			
Model 3					0.490	4.497 *
B	19.745 ***	0.219 ***	−0.143 *	−0.482 *		
SE B		0.865	−0.263	−0.233		

* *p* < 0.05, ** *p* < 0.01, *** *p* < 0.001.

**Table 5:** Regression models used to examine the correlations of the monthly incidence of sICH with air pollutants in different age groups.

Factor	Constant	PM2.5	O_3_	SO_2_	NO_2_	Adj. R^2^	F for Change in R^2^
Less than 25 years of age							
Model	X	X	X	X	X		
Between 25 and 44 years							
Model		X	X	X		0.101	7.616 **
B	1.711 ***				0.036 **		
SE B					0.341		
Between 45 and 64 years							
Model			X	X	X	0.380	37.195 ***
B	6.450 ***	0.068 ***					
SE B		0.625					
Between 65 and 79 years							
Model 1			X	X	X	0.474	54.077 ***
B	3.805 ***	0.071 ***					
SE B		0.695					
Model 2			X		X	0.554	11.527 **
B	4.559 ***	0.089 ***		−0.288 ***			
SE B		0.875		−0.346			
Model 3					X	0.583	4.938 *
B	5.643 ***	0.098 ***	−0.044 *	−0.310 ***			
SE B		0.957	−0.200	−0.372			
Model 4						0.607	4.377 *
B	6.384 ***	0.113 ***	−0.052 *	−0.290 ***	−0.059 *		
SE B		1.102	−0.236	−0.348	−0.228		
Over 80 years of age							
Model		X	X	X		0.211	16.746 ***
B	1.905 ***				0.059 ***		
SE B					0.473		

* *p* < 0.05, ** *p* < 0.01, *** *p* < 0.001.

## References

[1] Ohwaki K., Yano E., Murakami H., Nagashima H., Nakagomi T. (2004). Meteorological factors and the onset of hypertensive intracerebral hemorrhage. Int. J. Biometeorol..

[2] Liu Y., Goodson J.M., Zhang B., Chin M.T. (2015). Air pollution and adverse cardiac remodeling: Clinical effects and basic mechanisms. Front. Physiol..

[3] Vieira S.E. (2015). The health burden of pollution: The impact of prenatal exposure to air pollutants. Int. J. Chronic Obstr. Pulm. Dis..

[4] Tsai S.S., Goggins W.B., Chiu H.F., Yang C.Y. (2003). Evidence for an association between air pollution and daily stroke admissions in Kaohsiung, Taiwan. Stroke.

[5] World Health Organization (WHO) Ambient (Outdoor) Air Quality and Health. http://www.who.int/mediacentre/factsheets/fs313/en/.

[6] Qureshi A.I., Mendelow A.D., Hanley D.F. (2009). Intracerebral haemorrhage. Lancet.

[7] Van Asch C.J., Luitse M.J., Rinkel G.J., van der Tweel I., Algra A., Klijn C.J. (2010). Incidence, case fatality, and functional outcome of intracerebral haemorrhage over time, according to age, sex, and ethnic origin: A systematic review and meta-analysis. Lancet Neurol..

[8] Chan C., Ting H., Huang H. (2014). The incidence, hospital expenditure, and, 30 day and 1year mortality rates of spontaneous intracerebral hemorrhage in Taiwan. J. Clin. Neurosci..

[9] Xiang H., Mertz K.J., Arena V.C., Brink L.L., Xu X., Bi Y., Talbott E.O. (2013). Estimation of short-term effects of air pollution on stroke hospital admissions in Wuhan, China. PLoS ONE.

[10] Andersen Z.J., Kristiansen L.C., Andersen K.K., Olsen T.S., Hvidberg M., Jensen S.S., Ketzel M., Loft S., Sørensen M., Tjønneland A. (2012). Stroke and long-term exposure to outdoor air pollution from nitrogen dioxide: A cohort study. Stroke.

[11] Han M.H., Yi H.J., Kim Y.S., Kim Y.S. (2015). Effect of seasonal and monthly variation in weather and air pollution factors on stroke incidence in Seoul, Korea. Stroke.

[12] Turin T.C., Kita Y., Rumana N., Nakamura Y., Ueda K., Takashima N., Sugihara H., Morita Y., Ichikawa M., Hirose K. (2012). Ambient air pollutants and acute case-fatality of cerebro-cardiovascular events: Takashima Stroke and AMI Registry, Japan (1988–2004). Cerebrovasc. Dis..

[13] Chan C.L., Lin W., Yang N.P., Huang H.T. (2013). The association between the availability of ambulatory care and non-emergency treatment in emergency medicine departments: A comprehensive and nationwide validation. Health Policy.

[14] Lin K.B., Lai K.R., Yang N.P., Wu K.S., Ting H.W., Pan R.H., Chan C.L. (2015). Trends and outcomes in the utilization of laparoscopic appendectomies in a low-income population in Taiwan from 2003 to 2011. Int. J. Equity Health.

[15] Chan C., Ting H., Huang H. (2014). The definition of a prolonged intensive care unit stay for spontaneous intracerebral hemorrhage patients: An application with national health insurance research database. BioMed Res. Int..

[16] (2003). Introduction to the National Health Insurance Research Database (NHIRD). http://w3.nhri.org.tw/nhird/.

[17] (2017). Taiwan Government Open Data Platform. http://data.gov.tw/.

[18] Stavem K., Hoel H., Skjaker S.A., Haagensen R. (2017). Charlson comorbidity index derived from chart review or administrative data: Agreement and prediction of mortality in intensive care patients. Clin. Epidemiol..

[19] Chan C.L., You H.J., Huang H.T., Ting H.W. (2012). Using an integrated COC index and multilevel measurements to verify the care outcome of patients with multiple chronic conditions. BMC Health Serv. Res..

[20] (2013). Innovation Center for Big Data and Digital Convergence, Yuan Ze University. http://www.innobic.yzu.edu.tw/.

[21] Xing Y.F., Xu Y.H., Shi M.H., Lian Y.X. (2016). The impact of PM_2.5_ on the human respiratory system. J. Thorac. Dis..

[22] Wang Y., Eliot M.N., Wellenius G.A. (2014). Short-term changes in ambient particulate matter and risk of stroke: A systematic review and meta-analysis. J. Am. Heart Assoc..

[23] Chan C.C., Chuang K.J., Chien L.C., Chen W.J., Chang W.T. (2006). Urban air pollution and emergency admissions for cerebrovascular diseases in Taipei, Taiwan. Eur. Heart J..

[24] Villeneuve P.J., Chen L., Stieb D., Rowe B.H. (2006). Associations between outdoor air pollution and emergency department visits for stroke in Edmonton, Canada. Eur. J. Epidemiol..

